# Determinants of consumer attitudes and re-purchase intentions toward direct-to-consumer (DTC) brands

**DOI:** 10.1186/s40691-020-00224-7

**Published:** 2021-01-25

**Authors:** Naeun Lauren Kim, Daeun Chloe Shin, Gwia Kim

**Affiliations:** 1 Assistant Professor, Department of Design, Housing, and Apparel, College of Design, University of Minnesota, St. Paul, MN 55108 USA; 2 Ph.D. Student, Department of Textile and Apparel, Technology and Management, Wilson College of Textiles, North Carolina State University, Raleigh, NC 27606 USA

**Keywords:** Direct-to-Consumer, Digital retailing, E-commerce, Innovativeness, Cost-effectiveness

## Abstract

In the fashion and retail industry, a group of startups, referred to as Direct-to-Consumer (DTC) brands, are proliferating. DTC brands are defined as e-commerce brands that sell directly to consumers, without retailer ‘middlemen’ like department stores. They typically begin as a purely online business, fully leveraging digital channels for marketing and selling. Given the limited research on the topic, this paper aims to identify determinants of consumers’ attitudes and re-purchase intentions toward DTC brands. The initial qualitative phase of in-depth interviews with frequent DTC shoppers, resulted in the identification of eight determinants. The subsequent quantitative analysis with 210 US DTC shoppers confirmed that *co*-*creation*, *cost*-*effectiveness*, *website attractiveness*, *brand uniqueness*, *social media engagement*, and *innovativeness* of DTC brands significantly influence consumers’ attitudes while *cost*-*effectiveness* (indirectly), *brand uniqueness*, *social media engagement*, and *brand innovativeness* affect consumers re-purchase intentions. The findings offer insights for aspiring entrepreneurs and incumbent retailers on strengthening their value propositions.

## Introduction

The recent decline of the retail industry that manifested with retail bankruptcies and store closures was so spectacular that it was dubbed the retail apocalypse. Store closures by the end of 2019 exceeded the number of store closures in all of 2018, reaching more than 9000 in the U.S. (Unglesbee 2019). Retail bankruptcies increased by 35% in 2019, amounting to 23, and among the companies that filed for bankruptcy was Forever 21, an apparel brand that popularized fast fashion, of which business was still booming in 2015, with $4.4 billion in revenue (Thomas, 2019a; Wang and Kim 2019). J. Crew Group also ended up filing for bankruptcy in early 2020 (Isidore and Meyersohn 2020). This trend is expected to continue with about 75,000 store closures estimated in the U.S. by 2026 (Thomas 2019b). This change in the retail landscape has been mainly attributed to the rise of e-commerce and the decline of traditional retailers, caused by their primarily reliance on brick-and-mortar stores as a sales channel (Thomas 2019b).

Amidst the massive store closures, a group of digitally native startups referred to as Direct-to-Consumer (DTC) brands are maintaining a strong growth rate, higher than that of the total U.S. ecommerce sales, despite some DTC brands’ recent struggles due to overcrowding and increased customer acquisition costs (Lipsman 2020). These startups typically start as a purely online business, fully leveraging digital channels for marketing and sales, hence digitally native brands. They are DTC brands because they sell directly to consumers, without intermediaries or retailer ‘middlemen’ like department stores. What differentiate these brands from traditional online brands are their specialization on a single or small suite of related products, and innovations in the product or business model (Jin and Shin 2020). With a steady loyal customer base, these brands are establishing a foothold in the market (Zia 2017). A prime example is the clothing retailer Everlane. Since 2010, the brand has been growing exponentially, and in 2018, alongside Apple and Amazon, it was listed as one of TIME’s 50 Most Genius Companies (Time 2018).

The growing venture capital investments and the incumbent retailers’ acquisition of these startups attest to the DTC brands’ growth potential and value in the market. By 2017, investment deals involving DTC companies increased to 196, up more than 600% from 32 in 2010, including the $25 million Series B round (Chen 2019) of Reformation, an apparel brand founded in 2009. Recognizing the value of the DTC brands’ digital capabilities, as well as their appeal to consumers in the increasingly digitally driven market, high-profile incumbent retailers have been acquiring DTC companies. For example, in 2018, Walmart acquired ELOQUII, a women’s plus-size apparel brand founded in 2011, in addition to two other apparel DTC brands Walmart acquired in the previous year (Walmart 2018). In 2019, Wacoal International Corporation acquired Lively, a lingerie DTC brand founded in 2016 (Clark 2019). The DTC brands’ focus on digital channels has become ever more relevant, given the recent surge in online shopping, in response to the COVID-19 pandemic (Briedis et al. 2020).

The DTC brands’ continued growth despite the recent industry-wide struggles indicates much potential for the struggling incumbents to learn from these brands. However, till date no study has investigated what draws consumers to the DTC brands. While there is a significant body of prior research examining the drivers of positive online shopping outcomes, the dominant constructs of prior empirical models, such as online shopping quality (e.g., Ha and Stoel 2012) and e-service quality (Finn et al. 2009), are inadequate in fully capturing new online brand values, such as unique brand identity and brand innovativeness, that have arisen from the DTC brands’ product and business model innovations. Furthermore, previous studies on DTC brands in the retail industry is extremely limited and lacks empirical findings, as they mostly aimed to offer a conceptual overview of the DTC business model, such as its challenges and DTC brands’ branding strategies (Gielens and Steenkamp 2019), or explain the business model’s benefits from the company’s perspective (Jin and Shin 2020). To fill this research gap, this paper aims to empirically identify the determinants of the attitude and re-purchase intention toward DTC brands. The findings from this research will offer valuable business insights for not only the incumbents, but also aspiring entrepreneurs. For the incumbent retailers facing increasing competition from DTC brands, the findings can help them identify gaps in their current offerings, and ultimately strengthen their own value propositions. For aspiring entrepreneurs, whether in the early stage of brainstorming business ideas or in the final stage of refining them, the findings can serve as a competitive landscape, useful for developing and fine-tuning business ideas.

## Methods

### Qualitative phase method

Due to the limited availability of literature, a qualitative phase was first conducted to uncover the potential determinants of consumers’ DTC shopping attitudes and intentions. A semi-structured, in-depth interview approach was utilized, as it allows researchers to go beyond a surface understanding of a phenomenon (Kvale 1983). A total of six participants were recruited at a university in the Midwest U.S., using a purposive sampling method. Four female and two male participants were recruited with an average age of 22.6, and the ethnicity of the participants were White/Caucasian (4), Hispanic (1), and Asian (1). In order to understand what motivates consumers to continuously shop from DTC brands, only the frequent shoppers (e.g., those who have shopped from multiple DTC brands or have made repeat purchases from a DTC brand) were recruited. The participants were frequent shoppers from the following DTC brands: Away, Glossier, Everlane, Allbirds, Reformation, and Warby Parker.

Each interview lasted for about 30 min, and the interview questions included: “What aspects about the brand/product do you particularly like?” “Why did you choose this brand over similar others?” Data collection continued until data saturation was reached, meaning that by the sixth interviewer, no new information was discovered. Recordings of the interviews were transcribed and organized for further analysis following the general procedures of thematic analysis (Braun and Clarke 2006). Three researchers analyzed the data inductively by identifying patterns using open coding. Codes were created as researchers labelled participants’ answers that commonly emerged, which were then organized into different themes. Validity of the analysis was established through the peer evaluation process as more than one researcher conducted the analysis and corroborated the results (Lincoln and Guba 1985).

Specifically, the theme of co-creation was insinuated by two interviewees (Away and Glossier) with phrases such as “customer making an impact on the business,” “frequent communication,” “personalization.” Cost-effectiveness was alluded by all six interviewees with phrases including “affordable price” and “cheaper than traditional brands.” Website attractiveness was identified by the answers of Everlane and Glossier shoppers, “the website looks clean/nice.” Sustainability theme was created as the brand’s commitment to “environmental sustainability”, “transparent business practice,” “use of natural ingredients” were echoed by Reformation, Everlane, and Glossier shoppers respectively. All interviewees mentioned the uniqueness of the brand and its products, such as unique “style”, “design”, and “packaging”, which were collapsed into the brand uniqueness theme. The theme of social media engagement was identified after all shoppers cited social media as the main channel of brand’s communication and where they were first introduced to the brand. Finally, the innovativeness theme emerged as Warby Parker and Away shoppers described the brands’ “cool technology” and “features not available in traditional brands.” In addition to the interview, a review of pertinent trade articles and industry reports was conducted to further support the identified determinants.

### Determinant identification and hypotheses development

We identified eight determinants of the attitudes toward DTC brands, and of re-purchase intention from the qualitative phase: co-creation, cost-effectiveness, website attractiveness, sustainability, brand uniqueness, social media engagement, and brand innovativeness. Table 1 describes each determinant with examples.

**Table 1:** DTC Brand characteristics and examples

Characteristics	Descriptions	Examples
Co-creation	Process of building experiences and resolving problems with joint efforts by customers and brands	Everlane: Reflecting customers’ feedback to change materials and redesign details of items Glossier: Building the brand based on conversations the founder had with influential female figures on beauty products
Cost-effectiveness	Unique business model to offer high quality products at reasonable prices without the presence of middlemen	Warby Parker: Selling eyeglasses between $95 and $145 which is lower than the average price of branded eyeglasses ($263) Gymshark: Selling products between $25 and $60 which is lower than high-end alternative fitness clothing competitor brands
Website attractiveness	A website’s sole store front role to communicate brand identity and retain customers through thoughtfully designed website interfaces, such as icons, colors, graphics, music, and page lengths	Everlane: Website as only store front, which is perceived clean and aesthetic to consumers; highlights the brand’s sustainability efforts M. Gemi: Website entrance with a short clip that shows the brand’s association with Italian craftmanship
Sustainability	Eco-friendly and social activities embedded into a core of brand concept; providing transparent information about supply chain and pricing policies	Reformation: Accentuating the brand’s sustainability efforts through proactively sharing environmental impact of products and reporting on sustainability initiatives Allbirds: Brand identity rooted in sustainability, such as measuring the environmental impact of their products, using natural and recycled materials, and funding external sustainability projects
Brand uniqueness	Unique products and brand stories to differentiate from traditional brands; emphasis placed on the brand origin; product function/design specialization in niche categories	Bonobos: Offering uniquely designed product (e.g., pants that conform to the natural shape of the waist for comfort) Away: Minimalistic product designs with unique features like phone charging batteries
Social media engagement	Social media often the primary channel for marketing; hyperactive brand-customer interaction through social media; customer’s behavioral manifestations in social media beyond purchase as an online community	Glossier: 1) Brand’s Instagram and YouTube offers information of the brand and products 2) Consumers’ sharing experience of products in the online community Gymshark: 1) Turned fitness influencers into brand ambassadors 2) User generated contents, such as work-out videos, were shared on its YouTube channel to motivate other users.
Innovativeness	Innovative business model, products, and brand storytelling in which consumers perceive brands as being able to provide new and useful solutions to their needs; incorporation of cutting-edge technology (e.g., augmented reality, virtual try-on)	Warby Parker: Named on the list of innovative companies, highlighting technology of virtual try-ons

Developed by authors based on literature review and interviews

The first determinant is co-creation, which is the process of building experiences and resolving problems with joint efforts by customers and brands (Payne et al. 2008). Through direct interactions with customers, brands create ample opportunities to jointly create values through customized or co-produced offerings. As DTC brands interact directly with consumers, they can closely monitor customer feedback, and incorporate it into product development, allowing customers to co-create value. For instance, Everlane, a women’s and men’s apparel brand founded in 2010, to reflect customer feedback, changed the materials used for wool trousers to make them less itchy, and redesigned them by adding details such as belt loops and interior closures, as requested by their customers (Avins 2016). For Glossier, a beauty brand founded in 2014, co-creation was embedded into the brand identity from its inception. The brand was built from insights gained from the hundreds of conversations the founder of Glossier had with influential female figures, such as fashion models and businesswomen, to profile their makeup cabinets and share their beauty tips on her blog, ‘Into the Gloss’ that the founder started in 2010 (Glossier n.d.).

The interviews revealed that Glossier customers perceived the brand as attentive to customer opinions: “they’re very into what the consumers say, even on just little things.” An interviewee shared how she noticed on several occasions that improvements were made to the products based on customer feedback: “I got a lipstick kind of thing and they would fall out and it was cheap. So I figured a lot of people did that [would have a similar issue] and they changed the packaging for it.” Unlike a firm-centric approach, co-creation is customer-centric because customers are treated as active contributors to the development of product and service offerings, as opposed to passive recipients. As a result, the co-creation process highlights the customer’s point of view, and reflects their needs and wants, which often yields superior customer experiences (Prahalad and Ramaswamy 2004). It was shown that co-creation leads to positive brand evaluations (van Dijk et al. 2014). For example, van Dijk et al. (2014) showed that customers perceived brands that offer co-creation opportunities as more authentic and sincere. In addition, co-creation was also shown to increase purchase intention (See-To and Ho 2014), and behavioral loyalty (Cossío-Silva et al. 2016). Thus,

#### H1:

Perceived co-creation will positively influence (a) the attitude toward DTC brands and (b) the DTC re-purchase intention.

Without middlemen and physical stores, DTC brands enjoy higher margins, and thus are able to offer high quality products at reasonable prices. Thus, cost-effectiveness is one defining competitive advantage of DTC brands. Warby Parker, an eyeglasses retailer founded in 2010, offers eyeglasses priced between $95 and $145, significantly lower than the average price of $263 (Knowledge@Wharton 2013). Gymshark, a fitness clothing brand founded in 2012, is known for leggings with flattering shades, and they are priced between $25 and $60, significantly lower than high-end alternatives, which typically cost over $100 (Leighton 2018).

Cost-effectiveness means that offerings are “good value for the money” (Oliver and DeSarbo 1988). That is, DTC brands’ offerings have high perceived value or “consumer’s overall assessment of the utility of product (or service) based on perceptions of what is received and what is given”, relative to alternative offerings (Zeithaml 1988, p. 14). It also means that they are not necessarily the cheapest but better options, as shown by a Glossier customer’s comment: “I still prefer Glossier, but it’s a little bit higher price point.” Another interviewee’s comment revealed how the trade-off between price and quality is low for Glossier: “It’s really high quality for not super expensive stuff.” This view was shared by customers of other DTC brands like Warby Parker: “They’re way cheaper than any glasses that you can find” but “I’ve never seen that they were lower quality than any other glasses I bought.” Similarly, an Everlane customer shared that “their materials are really nice just in general, and I said this before, they are really high-quality even for just a basic t-shirt,” but “they are not that much more expensive.” The same view was echoed by a customer of Away, a luggage brand founded in 2015: “Design was my primary concern when choosing my luggage brand, and with a similar design, then the durability and the material, Away offered the cheapest price.” The interviews revealed that cost-effectiveness was one of the most cited reasons for patronizing DTC brands. This finding is consistent with previous empirical findings that showed perceived value’s positive influence on brand attitude (Alden et al. 2013), customer satisfaction, and willingness to pay (Li et al. 2012). Perceived value was also shown to increase purchase intention (Dall’Olmo Riley et al. 2015) and the behavioral intention of loyalty (Gounaris et al. 2007). Hence,

#### H2:

Perceived cost-effectiveness will positively influence (a) the attitude toward DTC brands and (b) the DTC re-purchase intention.

Since most DTC brands operate purely online, websites are their only storefront. The role of a physical store’s aesthetic design on shaping customer expectations and experiences is akin to a website’s ability to attract and retain customers through thoughtfully-designed website interfaces, such as icons, colors, graphics, music, and page lengths (Eroglu et al. 2001; Hausman and Siekpe 2009). Thus, the websites are expected to play a big role in shaping the consumer perception of the brand, as shown by an Everlane customer’s comment: “I really like the aesthetics of the brand and just even the way their website is laid out, and it’s super clean.” Another interviewee who also shopped with Everlane echoed this view: “I really like their website and their interface looks clean.” Websites also help DTC brands convey their unique brand identity to consumers. For example, Everlane’s website entrance emphasizes its sustainability and transparency efforts. Similarly, M.Gemi, an Italian shoe DTC brand, features a short clip showing its brand story of Italian craftmanship. Previous findings showed that aesthetically pleasing websites leads to more favorable brand attitude (Porat and Tractinsky 2012). Website attractiveness impacts subsequent consumer behaviors, leading customers to revisit the website (Rosen and Purinton 2004), make a purchase (Gregg and Walczak 2010), and repeat the purchase (King et al. 2016). Taken together:

#### H3:

Perceived website attractiveness will positively influence (a) the attitude toward DTC brands and (b) the DTC re-purchase intention.

Globally, corporate, social, and environmental sustainability have become increasingly an important criterion in consumer purchasing decisions. According to a Nielsen report, 73% of the respondents of a global survey said that they would definitely change their consumption habits to reduce their environmental impact (Nielsen 2018). Cognizant of this demand, quite a few DTC brands embed sustainability into their businesses. Reformation, an apparel brand founded in 2009, tracks the environmental impact of each product in terms of water usage, CO_2_ emissions, and wastes, and shares this information on their website. In addition, the brand provides detailed information about all their sustainability initiatives, from using energy-efficient lighting and appliances in the offices, to partnerships with non-profit organizations dedicated to protecting the environment (Reformation n.d.). Allbirds, a footwear company founded in 2014, takes a similar approach of measuring the environmental impact of their products, reducing the impact through the usage of natural and recycled materials, and offsetting the impact by funding external sustainability projects (Allbirds n.d.). The interviews revealed strong associations between some DTC brands and sustainability. A Reformation customer felt that “most of [DTC brands] have more of a mission connected with them. So if you’re going to buy from Reformation, you know that it is sustainable.” A similar view was shared by an Everlane customer: “[Everlane’s products] are made ethically which I really like. Everlane puts the factories in which the items are produced on all of their things. You can actually look them up and the procedures that they go through.” Empirical findings showed that more favorable attitudes toward socially responsible products lead to a higher willingness to pay (Ha-Brookshire and Norum 2011). It was also found that consumers have a higher purchase intention toward companies with better environmental performance (Grimmer and Bingham 2013).

Of course, DTC brands are not the only companies that promote sustainability. However, unlike traditional retailers, for DTC brands, sustainability is built into their businesses at their inception and at the core of their marketing and branding, all of which lend more credibility and authenticity to their sustainability claims. DTC brands’ credibility and authenticity are expected to negate the increasing consumer skepticism toward corporate sustainability claims (do Paço and Reis 2012). Previous findings showed that if consumers perceive the company’s sustainability initiatives to be intrinsically motivated, they evaluate the company more favorably (Parguel et al. 2011). Similarly, if consumers perceive the company’s commitment to sustainability more credibly, they have more favorable attitudes towards the brand (Olsen et al. 2014), and higher purchase intention (Leonidou and Skarmeas 2017). Therefore,

#### H4:

Perceived sustainability will positively influence (a) the attitude toward DTC brands and (b) the DTC re-purchase intention.

Consumers’ perceived brand uniqueness refers to “the degree to which customers feel the brand is different from competing brands—how distinct it is, relative to competitors” (Netemeyer et al. 2004, p. 211). With a strong point-of-difference, consumers can easily notice, recognize, and recall a brand over other competing brands (Netemeyer et al. 2004). Well-positioned DTC brands compete with other brands by differentiating their product offerings (CB Insights 2019). Bonobos focuses on men’s pants with signature curved waistbands that conform to the natural shape of the waist for comfort (Bonobos n.d.). Similarly, Allbirds is known for their unique woolen running shoes, made of eco-friendly and machine-washable materials, specifically designed for sockless wear (Allbirds n.d.). As such, the DTC brands established their niche positions by offering uniquely designed products, compared to traditional brands (CB Insights 2019). Interview respondents commented on why they purchase DTC products: uniqueness. For Allbirds’ product, one said that “I knew I didn’t want like normal shoes. I wanted something that was unique. That is why I really like them [Allbirds]”. Similarly, for Warby Parker, “They’re more stylish and more unique I would say, than most glasses you can find”.

In addition to the unique product design, DTC brands differentiate themselves from traditional retailers by highlighting their unique brand stories. For example, several DTC brands offer digital content that creates brands’ stories on their websites, such as the origin of brands and sustainability efforts (Martin 2020). M.Gemi has incorporated the Italian craftsmanship concept behind its origin and brand story (Gemi n.d.). Glossier shared the owner’s entrepreneurial story behind the brand’s birth (Glossier n.d.), and the story has given the brand its unique identity. Such sentiment was echoed during the interview. One respondent shared how she was impressed by the Glossier founder, Emily Weiss: “She’s a really good role model. I love learning about her. She supports women in business… And I think that’s cool. Just hearing her story it’s really inspirational… That’s kind of what I want to do. The way she persevered against people saying no.” Another respondent on the Glossier’s packaging that maintains the brands’ unique atmosphere: “I really liked the packaging that they have for Glossier. They have positive messages and they always put free makeup bags and stickers and samples and it’s in a really cute pink box… [The messages say] have a nice day, be you, just stay beautiful, those kinds of typical things but they’re still nice to see”. Previous literature found that the brand uniqueness is important for a brand to succeed (Netemeyer et al. 2004).

Consumers’ desire to differentiate themselves from others can be fulfilled by possessing unique products (Snyder 1992). Thus, when consumers purchase a unique product, the value of the product increases. As the value of the product increases, consumers’ perceived product uniqueness results in higher purchase intention (Wu et al. 2012). Similarly, unique brand positioning can lead to a sustainable competitive advantage, and a compelling reason to purchase the brand (Lassar et al. 1995). More specifically, brands with distinctive stories have higher brand trust (Schallehn et al. 2014), and brand uniqueness is shown to increase re-purchase intention, given greater brand equity (Lin et al. 2015). Therefore,

#### H5:

Perceived brand uniqueness will positively influence (a) the attitude toward DTC brands and (b) the DTC re-purchase intention.

Social media is a critical communication tool for DTC brands to interact with their consumers, conveying both promotional and informational messages, and offering a platform for their customers to communicate (Schlesinger et al. 2020). One example is Glossier. Information shared by the brand and its consumers through social media has helped increase the brand and product awareness (Danziger 2018). One interviewee stated about Glossier: “I followed them on Instagram and I got all the information from Instagram.” She continued to say that she learns ideas on how to use Glossier products from social media: “I remember watching these YouTube videos, get ready with me and they were kind of shows [that teach] you how to use their products… And then when they come out with new launches, they have a really good way of marketing it and showing different ways to use them. So it makes me actually really want to buy it.” Glossiers’ consumers within the online community also guided her to become more interested in Glossier products. The fellow consumers’ experiences along with Glossier’s own advertisements create synergy: “I saw someone else have a really full lip with gloss and then I was like, oh that looks really nice. So I asked them about it and they said Glossier. And then, after that I saw more advertisements on Instagram and Twitter. Because all of the Instagram pictures and the marketing looked really nice and just natural and glossy.” Another example is Gymshark as the brand turned existing fitness influencers into their brand ambassadors. These ambassadors partake in brand storytelling by sharing work-out videos through YouTube channels to motivate other users (Gilliand 2019). These social media engagement behaviors, which are a customer’s behavioral manifestations in social media beyond purchase (Dolan et al. 2019), occur actively among DTC brands’ consumers. The online community serves as a virtual space where the community’s members can also share friendship, recreation, common interests, and social support, as well as information (Ridings and Gefen 2004; Wasko and Faraj 2000). Consumers who form a community and receive information, desire to continue these relationships and are likely to commit to the brand more (Jin et al. 2010). Direct relationships between social media communications, whether they are firm-created or user-created messages, and brand attitude have been recognized in prior research (Časas et al. 2016; Ho and Wang 2015). Additionally, consumers who participated in an online community have shown greater repurchase intentions for the brand (Schivinski and Dabrowski 2016). Thus,

#### H6:

Perceived social media engagement will positively influence (a) the attitude toward DTC brands and (b) the DTC re-purchase intention.

Brand innovativeness is “the extent to which consumers perceive brands as being able to provide new and useful solutions to their needs” (Eisingerich and Rubera 2010, p. 66). Based on a unique business model of directly reaching consumers, DTC brands provide distinctive features that are not typically offered by traditional brands (CB Insights 2019). The innovation can include business models, products, storytelling, and all the other brand activities (Schlesinger et al. 2020). Indeed, Warby Parker and Everlane have been named on the list of Fast Company’s “World’s Most Innovative Companies” (Fast Company 2016). The interviews confirmed that consumers perceive that DTC brands are innovative, especially in terms of technology. For example, a Warby customer highlighted the emerging technology of virtual try-ons, and how this is the best part of the brand: “It’s really cool to see all of the features they have on their website. You can take a picture of your face and try them on virtually. I think the best part of the brand is that you can do the trial before you actually commit to buying them.”

Earlier studies revealed that greater consumers’ perceived brand innovation leads to positive consumers’ responses, such as consumer satisfaction, brand loyalty, and brand credibility (Pappu and Quester 2016; Shams et al. 2017). It was also found that as perceived brand innovativeness enhances brand credibility, it can subsequently increase desire to purchase (Shams et al. 2017). Furthermore, when consumers consider the brand to be innovative, they are likely to form positive attitudes towards the brand (Sanayei et al. 2013). Accordingly,

#### H7:

Perceived brand innovativeness will positively influence (a) the attitude toward DTC brands and (b) the DTC re-purchase intention.

In the context of online shopping, previous findings showed a positive relationship between attitudes and re-purchase intentions (Bupalan et al. 2019; Choo and Park :^2013^; Jiménez and San-Martín 2017). As such, it is reasoned that consumers with favorable attitudes toward DTC brands are more inclined to buy from DTC brands again. More formally,

#### H8:

Attitude toward DTC brand will positively influence the DTC re-purchase intention.

### Quantitative phase method

The purpose of the quantitative phase was to test the hypothesized relationships that were developed in the qualitative phase. Data was collected from a total of 210 U.S. consumers aged 18 years and older, via a professional online survey company. A stratified sampling method was used to ensure even representation of age and gender groups. A list of 20 popular fashion DTC brands were shown to respondents, and only those who have previously purchased from one or more DTC brands participated in the survey. When answering the survey, respondents were asked to recall their shopping experience with the DTC brands. The respondents’ demographic information is provided in Table 2.

**Table 2:** Sample demographics

Variable	N	%
Gender		
Male	105	50.0
Female	105	50.0
Age		
18–25	34	16.2
26–35	49	23.3
36–45	51	24.3
46–55	34	16.2
> 56	42	20
Education		
High school or less	29	13.8
College	139	66.2
Graduate School	42	20.0
Individual income		
< $20,000	22	10.5
$20,001–$40,000	39	18.6
$40,001–$60,000	45	21.4
$60,001–$80,000	32	15.2
$80,001–$100,000	21	10.0
> $100,001	51	24.3

The latent variables were measured by using multi-item scales that were adapted from previous studies. The measurement items are summarized in Table 3. All of the items were measured using seven-point Likert-type scales (1 = strongly disagree and 7 = strongly agree). In order to reduce the issue with common method bias, the independent and dependent variables were presented separately in random order, reducing the possibility of detecting patterns and subjectively responding to variables (Gabrielsson et al. 2012). Since the scope of this research was to mainly measure the significance and relative strength of the determinants on the two endogenous variables, the analysis did not assess the interdependencies between the determinant variables.

**Table 3:** Measurement items and exploratory factor analysis results

Items	Factor loadings	AVE	Composite reliability	Cronbach’s alpha
Co-creation (Cao et al. 2005)		.658	.906	.870
DTC brands have interactive feedback mechanism between customer and business	.793			
DTC brands offer personalization features	.828			
DTC brands have empathy with customers’ problems	.810			
DTC brands are very concerned about my welfare	.809			
DTC brands allow me to provide direct input to the brand	.819			
Cost-effectiveness (Fornell et al. 1996; Lamberton and Rose 2012)		.846	.916	.820
For the given price, I rate the DTC brand’s offer as good	.920			
For the given quality of the DTC brand’s product, I rate the price as good	.920			
Website attractiveness (Cao et al. 2005)		.718	.939	.921
I find the DTC brand’s website attractive	.770			
I find the DTC brand’s website appealing	.858			
I find the DTC brand’s website engaging	.879	.		
I find the DTC brand’s website gets me excited	.839			
I find the DTC brand’s website fun	.870			
I find the DTC brand’s website entertaining	.865			
Sustainability (Park and Kim 2016)		.721	.940	.923
DTC brand adopts environmentally friendly production practices	.837			
DTC brand’s clothes are produced with a minimum effect on the environment (e.g., no gases, low carbon footprint) and animals	.860			
DTC brand’s clothes are made from sustainable materials such as organic cotton and not be synthetic	.867			
DTC brand’s products are made under safe and healthy working conditions, without child labor or sweatshops	.842			
DTC brand pays fair wage for factory workers and raw material suppliers	.865			
DTC brand gives back to the communities in which it does business	.829			
Brand uniqueness (Franke and Schreier 2008)		.878	.956	.931
I perceive the DTC brand as highly unique	.930			
The DTC brand is one of a kind	.943			
The DTC brand is really special	.938			
Social media engagement (Baldus et al. 2015; Lamberton and Rose 2012)		.822	.970	.964
DTC brand’s social media is my critical connection for new and important information about the brand and its products	.893			
DTC brand’s social media keeps me on the leading edge of information about the brand	.915			
When I want up-to-date information about this brand, I look to DTC brand’s social media	.902			
DTC brand’s social media is the best way to stay informed about new developments with this brand	.903			
Engaging in DTC brand’s social media allows me to be part of a group of like-minded people	.913			
Engaging in DTC brand’s social media allows me to belong to a group of people with similar interests	.918			
Innovativeness (Kunz et al. 2011)		.680	.937	.921
DTC brand is dynamic	.812			
DTC brand is very creative	.836			
DTC brand launches new products and creates market trend all the time	.826			
DTC brand is a pioneer in its category	.818			
DTC brand constantly generates new ideas	.859			
DTC brand has changed the market with its offer	.794			
DTC brand is an advanced-forward looking firm	.827			
Attitude (Ajzen 1991)		.759	.926	.894
All things considered, I find shopping from DTC brands to be a wise move	.820			
All things considered, I think purchasing from DTC brands to be a positive thing	.893			
All things considered, I think shopping from DTC brands is a good thing	.883			
Overall, buying products from DTC brands makes sense	.888			
Re-purchase Intention (Bhattacherjee 2001)		.840	.955	.936
All things considered, I expect to continue purchase from DTC brand often in the future	.882			
I can see myself buying from DTC brand more frequently in the future	.928			
I can see myself increasing my purchase from DTC brand if possible	.938			
It is likely that I will frequently buy products from DTC brand in the future	.918			

## Results

### Reliability and validity

An exploratory factor analysis was performed using SPSS 25, and the results are summarized in Table 3. The indicator validity was checked with all factor loadings exceeding a recommended threshold of .70 (Hair et al. 2010). Internal consistency was confirmed with all constructs’ Cronbach’s alpha values, and composite reliability values exceeding .70. Convergent validity was established, as the average variances extracted (AVE) were all greater than the acceptable threshold of .5 (Bagozzi and Yi 1988). Finally, discriminant validity was confirmed by comparing the square roots of the AVE values with the corresponding estimates of the correlation values (Fornell and Larcker 1981) (Table 4). Overall, the measurement items fulfilled the reliability and validity requirements for further analysis.

**Table 4:** Correlation matrix

	Mean	SD	1	2	3	4	5	6	7	8	9
Co-creation	5.168	1.016	.*811*								
Cost-effectiveness	5.676	.940	.556	*.920*							
Website attractiveness	5.462	.993	.677	.582	*.847*						
Sustainability	5.102	1.038	.635	.434	.557	*.850*					
Brand uniqueness	5.378	1.191	.609	.543	.650	.606	*.937*				
Social media engagement	4.873	1.557	.542	.474	.678	.475	.572	*.907*			
Innovativeness	5.582	.936	.634	.546	.717	.620	.765	.632	*.824*		
Attitude	5.554	1.094	.587	.656	.646	.479	.513	.595	.593	*.871*	
Repurchase intention	5.708	.987	.577	.640	.636	.522	.614	.517	.594	.779	*.917*

The lower triangle of the matrix represents the correlation coefficients between constructs

The diagonal values (italics values) represent the square root of the average variance extracted of each construct

### Measurement model

The data was analyzed through the partial least squares path modeling technique (PLS-SEM), using SmartPLS 2.0 software. As a component-based modelling approach, PLS is often preferred to covariance-based approaches such as structural equation modelling (CB-SEM), and multiple regression when estimating a complex path model with a small sample (Chin 1998; Hair et al. 2010). Our sample size of 210 satisfied the criterion for PLS-SEM with 10 times the largest number of structural paths directed at a particular construct in the structural model, as the sample size threshold for our model would have been 90 (Hair et al. 2011). Moreover, the use of PLS modeling is recommended when the research model is exploratory in nature, rather than confirmatory (Hair et al. 2011). A nonparametric bootstrapping procedure was conducted to test the significance of path coefficients.

**Fig. 1:**
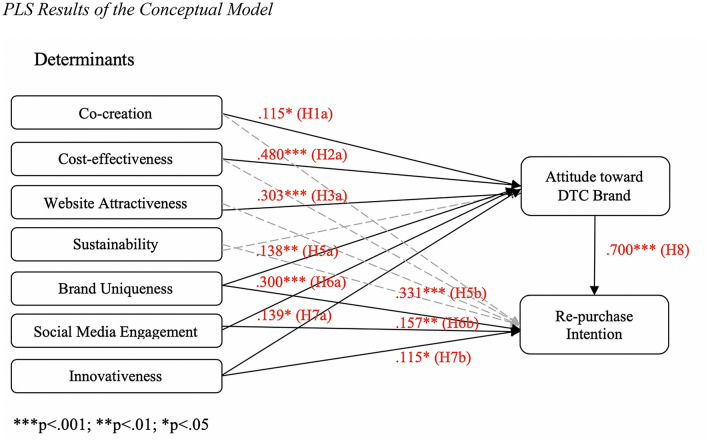
PLS results of the conceptual model

The results of the analysis are summarized in Fig. 1 and Table 5. The analysis reveals that the following variables significantly influenced the consumers’ *attitudes toward DTC brands*: co-creation (β = .115, p < .05), cost-effectiveness (β = .480, p < .001), website attractiveness (β = .303, p < .001), brand uniqueness (β = .138, p < .01), social media engagement (β = .300, p < .001), and innovativeness (β = .139, p < .01). Hence, H1a, H2a, H3a, H5a, H6a, and H7a were supported. Sustainability was the only variable that did not have a significant effect on attitude, rejecting H4a. On the other hand, the determinants that significantly influenced consumers’ *re*-*purchase intentions* include brand uniqueness (β = .331, p < .001), social media engagement (β = .157, p < .01), and innovativeness (β = .115, p < .01), supporting H5b, H6b, and H7b. The variable, co-creation (H1b), cost-effectiveness (H2b), website attractiveness (H3b), and sustainability (H4b), did not significantly affect re-purchase intentions. Additionally, the indirect effects of the independent variables on re-purchase intentions through attitudes were analyzed (see Table 6). While most findings did not differ significantly from the results of the direct effects, it was discovered that the cost-effectiveness variable had a significant indirect influence on re-purchase intentions through attitude (β = .335, p < .001). Finally, consumers’ attitudes toward DTC brands had a positive, and significant influence on their re-purchase intentions (β = .700, p < .001), which suggests a strong correlation between attitude and behavioral intentions (H8 supported).

**Table 5:** Results of the Hypothesis Testing

Hypothesis	Beta	Support
H1a. Co-creation → Attitude toward DTC brand	.115*	Yes
H1b. Co-creation → Re-purchase intention	− .025	No
H2a. Cost-effectiveness → Attitude toward DTC brand	.480***	Yes
H2b. Cost-effectiveness → Re-purchase intention	.114	No
H3a. Website attractiveness → Attitude toward DTC brand	.303***	Yes
H3b. Website attractiveness → Re-purchase intention	.082	No
H4a. Sustainability → Attitude toward DTC Brand	.072	No
H4b. Sustainability → Re-purchase intention	.096	No
H5a. Brand uniqueness → Attitude toward DTC brand	.138**	Yes
H5b. Brand uniqueness → Re-purchase intention	.331***	Yes
H6a. Social media engagement → Attitude toward DTC brand	.300***	Yes
H6b. Social media engagement → Re-purchase intention	.157**	Yes
H7a. Innovativeness → Attitude toward DTC brand	.139*	Yes
H7b. Innovativeness → Re-purchase intention	.115*	Yes
H8. Attitude toward DTC brand → Re-purchase intention	.700***	Yes

*** p < .001; ** p < .01; * p < .05

**Table 6:** Results of the indirect effects

Path	Beta	Support
Co-creation → Attitude → Intention	.077	No
Cost-effectiveness → Attitude → Intention	.335***	Yes
Website attractiveness → Attitude → Intention	.100	No
Sustainability → Attitude → Intention	.027	No
Brand uniqueness → Attitude → Intention	.231***	Yes
Social media engagement → Attitude → Intention	.147**	Yes
Innovativeness → Attitude → Intention	.118*	Yes

*** p < .001; ** p < .01; * p < .05

The percentage of variance explained by the predictors for the endogenous variable of attitude toward DTC brand was 58.0% (R^2^ = .580). The predictors for re-purchase intentions accounted for 68.7% of the variance (R^2^ = .687). These R^2^ values suggest that a high percentage of variance of the endogenous variables was explained, showing support for the conceptualized model.

## Discussion and conclusion

The findings reveal the role of various determinants in explaining consumers’ attitude and re-purchase intentions for DTC fashion brands. First, all variables (e.g., co-creation, cost-effectiveness, website attractiveness, brand uniqueness, social media engagement, and brand innovativeness) except *sustainability* were found to have a positive effect on attitude towards DTC brands. The insignificant influence of sustainability implies that consumers who are attracted to DTC brands are not drawn for the brands’ stainability efforts. This may be because while the consumers’ demand for sustainable business practices has been increasing, not all DTC brands have fully adopted such practices into their business model yet. Thus, the consumers’ experience with sustainable DTC products may have been limited. It also echoes the social desirability paradox that consumers say they want sustainable brands, but their purchase decisions often do not follow through (White et al. 2019). The magnitude of the standardized regression coefficients suggests that *cost*-*effectiveness* (beta = .480) carries greater relative importance than the other determinants. Such results align with the fact that DTC brands operate without any middlemen and physical stores, hence have the capability to offer high quality products at lower prices than traditional retailers. After cost-effectiveness, website attractiveness and social media engagement had stronger impact on attitude than other variables. In other words, respondents seem to be driven by the ability to engage with brands directly, whether through *social media* or *websites*, and subsequently *co*-*create* value. This highlights the DTC brands’ strong focus on customer relationships in order to connect every aspect of their business with consumers: from their website to their product, and every touchpoint in between. Particularly, through active engagement via social media, DTC brands have allowed for a mutual exchange of benefits, rather than one-way transactions (Schlesinger et al. 2020). Our findings further indicate that DTC brand’s *innovativeness* and *uniqueness,* in regard to their product offering and storytelling, enhances consumers’ perceptions of the brands. This illustrates DTC brands’ efforts to seek out the newest technology and designs to set themselves apart from mainstream competitors and establish a niche position in the market (CB Insights 2019).

On the other hand, the determinants of re-purchase intentions included *brand uniqueness*, *social media engagement*, *innovativeness, and cost*-*effectiveness* (indirect effect). While all of them have been previously identified as determinants of attitude toward DTC brands, the omission of certain variables (e.g., *co*-*creation*, *website attractiveness*) indicates that there are some discrepancies between reported attitudes and actual behavior of consumers. In other words, determinants such as co-creation and website attractiveness aid in establishing a positive attitude but do not necessarily lead consumers to choose DTC brands again. Although consumers may be initially enticed to the interactive communication of DTC brands and their charming web storefronts, these factors alone are not attractive enough to make a purchase. Rather, it was the brand uniqueness variable that exhibited the strongest influence on re-purchase intentions. This finding suggests that consumers’ behavioral intentions are mainly influenced by the DTC brands’ *branding efforts* to innovate and differentiate. In addition, cost-effectiveness was shown to indirectly influence re-purchase intentions through attitudes, confirming prior research on the role of cost-effectiveness on purchase decisions (Dall’Olmo Riley et al. 2015; Li et al. 2012). Such discovery further supports the value-driven business model of DTC brands and its appeal to consumers in building loyalty. Lastly, it was found that there is a strong association between consumers’ attitude and re-purchase intentions, in accordance with prior research (Glasman and Albarracín 2006).

### Theoretical and managerial implications

This is one of the first academic studies on DTC brands that explains the appeal of these rising startups. To stay relevant and stand out in the fast-moving and saturated market, online retailers have been continuously innovating themselves in various aspects: from product development (e.g., Everlane’s co-creation), and product delivery (e.g., Warby Parker’s virtual try-on), to brand-building (e.g., Glossier’s online community). However, current academic studies are insufficient in capturing new online brand values arising from these emerging innovations and strategies, and how they influence arguably one of the most important retail outcomes: re-purchase intentions or behavioral loyalty.

Previous e-commerce literature has focused on the most salient and basic factors related to online shopping, such as fulfillment capabilities (ease of ordering, on-time delivery), privacy and security, and online store design (website navigation, quality) (King et al. 2016; Reibstein 2002). The role of hard-to-imitate brand values, such as brand uniqueness, has received little attention in the e-commerce context, with a few exceptions (e.g., Fazal-e-Hasan et al. 2019). This underexplored consumer value (i.e., brand uniqueness), however, was shown to be the most important determinant of online behavioral loyalty, more so than social media engagement, which has received much attention from both academia (Dolan et al. 2019) and industry (Stanko et al. 2019). In addition, while previous research has demonstrated the role of co-creation (e.g., van Dijk et al. 2014), cost-effectiveness (e.g., Alden et al. 2013), website attractiveness (e.g., Porat and Tractinsky 2012), and social media engagement (e.g., Schivinski and Dabrowski 2016) on online consumers’ brand evaluations, their relative importance has been unclear. In fact, the findings of this study suggest that the influence of factors like co-creation, sustainability, and website attractiveness on consumers’ behavioral intentions are minimal relative to other determinants. Rather, consumers were shown to base their purchase decisions on the brands’ unique, innovative, and value-driven products. The current study’s value lies in not only identifying a comprehensive list of antecedents of online brand attitudes and behavioral loyalty, but also showing their relative contributions.

The study also generates managerial implications. The findings offer insights on how startup brands can develop or refine their business ideas for value positioning. Given that brand uniqueness and innovativeness were significant determinants of both positive consumer attitudes and re-purchase intentions, startups should offer unique values by challenging the status quo, and fulfilling the unmet needs of consumers through product and business model innovations. The fruition of innovations is demonstrated by successful DTC brands’ pioneering offerings: Warby Parker’s online distribution of less expensive eyeglasses, coupled with its virtual try-on and free home try-on services; Allbirds’s machine-washable and breathable wool shoes; and Away’s durable yet colorful luggages, equipped with a removable battery for charging electronic devices. In addition, given that cost-effectiveness was the strongest predictor of positive brand attitudes, leading to higher repurchase intentions, DTC startups are recommended to highlight their pricing advantages compared to traditional retailers’ higher mark-ups. Given that factors like co-creation and website attractiveness failed to influence re-purchase intentions, despite their positive effects on consumer attitudes, it is recommended that DTC brands place a greater emphasis on the aforementioned branding and differentiation efforts on a macro level, rather than on micro marketing strategies like clean websites.

Furthermore, incumbent retailers facing competition from DTC brands can reassess and strengthen their own value propositions. One lesson they can learn from DTC brands is to establish a unique brand identity, whether through specializing in a particular product category, producing creatively designed products instead of churning out cookie-cutter designs, or telling a unique story behind the brand. Additionally, given that social media engagement significantly predicted both positive consumer attitudes and repurchase intentions, the incumbents are recommended to pay particular attention to DTC brands’ capabilities for social media marketing and building a strong online brand community. The importance of such digital marketing and sales capabilities has heightened as customer traffic to digital channels, which surged after the COVID-19 pandemic, is expected to morph into a permanent change in consumer behavior (Briedis et al. 2020).

Unlike DTC brands that successfully leverage the digital channels for both customer acquisition and retention, many traditional retailers are ill-equipped to offset reduced foot traffic to physical stores, especially ones that prioritize physical stores and in-person engagement over omnichannel strategies. It is recommended that such incumbents prioritize digital channels over physical stores, redefine the role of physical stores, and adopt omnichannel models, such as one that allows customers to buy online and pick up in store. It is crucial that the retailers adopt effective strategies to more proactively lower the risk of shopping online. For example, to address the inability to try on items prior to making a purchase, consider offering a virtual try-on service, powered by advanced virtual and augmented reality technologies. It should be realistic enough to adequately replace an actual try-on (Kim and Forsythe 2008). It is also advised that the retailers integrate their digital channels for delivering seamless and consistent services and experiences. In short, to avoid falling into permanent irrelevance, the struggling incumbents should pivot their businesses by boosting digital presence and engagement.

Lastly, contrary to popular belief that incorporating sustainability into a business is a good practice (Nielsen 2018), our non-significant results suggest that placing it at the forefront of branding and marketing does not necessarily guarantee traction. This point is bolstered by the findings of prior studies that examined the relative importance of sustainability. For example, more tangible factors, such as price and fit, were routinely ranked higher than sustainability (Nilssen et al. 2019; Rothenberg and Matthews 2017). In short, while sustainability is an important factor, it is not consumer’s top priority. Therefore, it is recommended that brands keep in mind this important caveat that other consumer benefits should precede sustainability and prioritize the aforementioned consumer values shown to significantly predict consumer attitudes and re-purchase intentions.

### Limitations and suggestions for future research

The limitations of this study suggest directions for future research. First, the sample utilized in this study was of shoppers of twenty well-known DTC fashion brands. Consumers’ perceptions and experiences with DTC brands not listed in the survey, or non-users, may be different from those examined here. In order to strengthen the generalizability of the model, samples with other DTC brand shopping experiences and wider demographics are recommended. Second, further investigation is suggested to explore the deterrents of DTC shopping, and understand why consumers may prefer traditional retailers and feel hesitant to try out DTC brands, as doing so will help DTC brands identify areas of improvement. Third, future studies could explore the typology of DTC consumers. Understanding the characteristics and values of consumers beyond simple demographic information would help the industry and researchers better target consumers.

## Data Availability

The datasets used and/or analyzed during the current study are available from the corresponding author on reasonable request.
